# Incidence and Clinical Features of Early Stent Thrombosis in the Era of New P2y12 Inhibitors (PLATIS-2)

**DOI:** 10.1371/journal.pone.0157437

**Published:** 2016-06-16

**Authors:** Elad Asher, Arsalan Abu-Much, Ilan Goldenberg, Amit Segev, Avi Sabbag, Israel Mazin, Meital Shlezinger, Shaul Atar, Doron Zahger, Arthur Polak, Roy Beigel, Shlomi Matetzky, Platelets and Thrombosis in Sheba-PLATIS Study Group

**Affiliations:** 1 Leviev Heart Center, Sheba Medical Center, Tel Hashomer, affiliated to the Sackler Faculty of Medicine, Tel Aviv University, Tel Aviv, Israel; 2 Division of Cardiology, Galilee Medical Center, Nahariya, Israel; 3 Department of Cardiology, Soroka Medical Center, Beer Sheba, Israel; 4 Heart Institute, Hadassah University Hospital, Jerusalem, Israel; Medstar Washington Hospital Center, UNITED STATES

## Abstract

Early stent thrombosis (EST) (≤ 30 days after stent implantation) is a relatively rare but deleterious complication of percutaneous coronary intervention (PCI). Administration of newer P2Y12 inhibitors (prasugrel and ticagrelor) combined with aspirin has been shown to reduce the incidence of sub-acute and late stent thrombosis, compared with clopidogrel. We investigated the “real life” incidence of EST in patients from a large acute coronary syndrome (ACS) national registry, where newer P2Y12 inhibitors are widely used. Patients were derived from the ACS Israeli Survey (ACSIS), conducted during 2006, 2008, 2010 and 2013. Major adverse cardiac events (MACE) at 30days were defined as all-cause death, recurrent ACS, EST and stroke.Of the 4717 ACS patients who underwent PCI and stenting, 83% received clopidogrel and 17% newer P2Y12 inhibitors. The rate of EST was similar in both groups (1.7% in the newer P2Y12 inhibitor group vs. 1.4% in the clopidogrel-treated patients, p = 0.42). Results were consistent after multivariate analysis (adjusted HR = 1.06 [p = 0.89]). MACE occurred in 6.4% in the newer P2Y12 inhibitor group compared with 9.2% in the clopidogrel group (P<0.01). However, multivariate logistic regression modeling showed that treatment with newer P2Y12 inhibitors was not significantly associated with the secondary endpoint of MACE when compared with clopidogrel therapy [OR = 1.26 95%CI (0.93–1.73), P = 0.136]. The incidence of "real life" EST at 1month is relatively low, and appears to be similar in patients who receive newer P2Y12 inhibitors as well as in those who receive clopidogrel.

## Introduction

Early stent thrombosis (EST) (≤ 30 days after stent implantation) is a rare but severe complication which could present as ST-elevation myocardial infarction (STEMI) or sudden cardiac death within the first 30 days after stent implantation [1, 2]. EST is more common following stent implantation in the context of acute coronary syndrome (ACS) than in stable coronary disease, particularly in patients with multi-vessel disease and in those presenting with a Killip class of ≥2 [1–4]. This observation can be explained by platelet activation and a heightening of the coagulation process as part of the pathogenesis of ACS [5, 6]. Previous studies have shown that several patient-related variables are associated with EST during ACS, such as suboptimal antiplatelet administration, insulin-requiring diabetes, hypertension and baseline renal insufficiency [3–6], in addition to several other independent predictors, such as final stent minimal luminal diameter, non-administration of thienopyridine prior to percutaneous coronary intervention (PCI) and high baseline hemoglobin levels [5–7]. Newer antiplatelet medications, including ticagrelor [8] and prasugrel [9], are associated with a significant reduction in the incidence of late stent thrombosis (>30 days following stent implantation) and sub-acute stent thrombosis (> 24 hours but <30 days after stent implantation). However, neither drug showed reduction in acute stent thrombosis during the first 24 hours after stent implantation, when compared with clopidogrel [8–11], even when ticagrelor was administrated as part of a pre-hospital ACS regimen [12]. Nevertheless, data regarding the rate of EST in the new era of antiplatelet drugs are scarce. Hence, we decided to investigate the trend and incidence of EST in a large national ACS registry in a “real life” setting, where the administration of antiplatelet drugs prior to PCI is standard care, incorporating third generation drug-eluting stents and newer P2Y12 inhibitors (specifically, prasugrel and ticagrelor).

## Materials and Methods

### Study population

**Fig 1:**
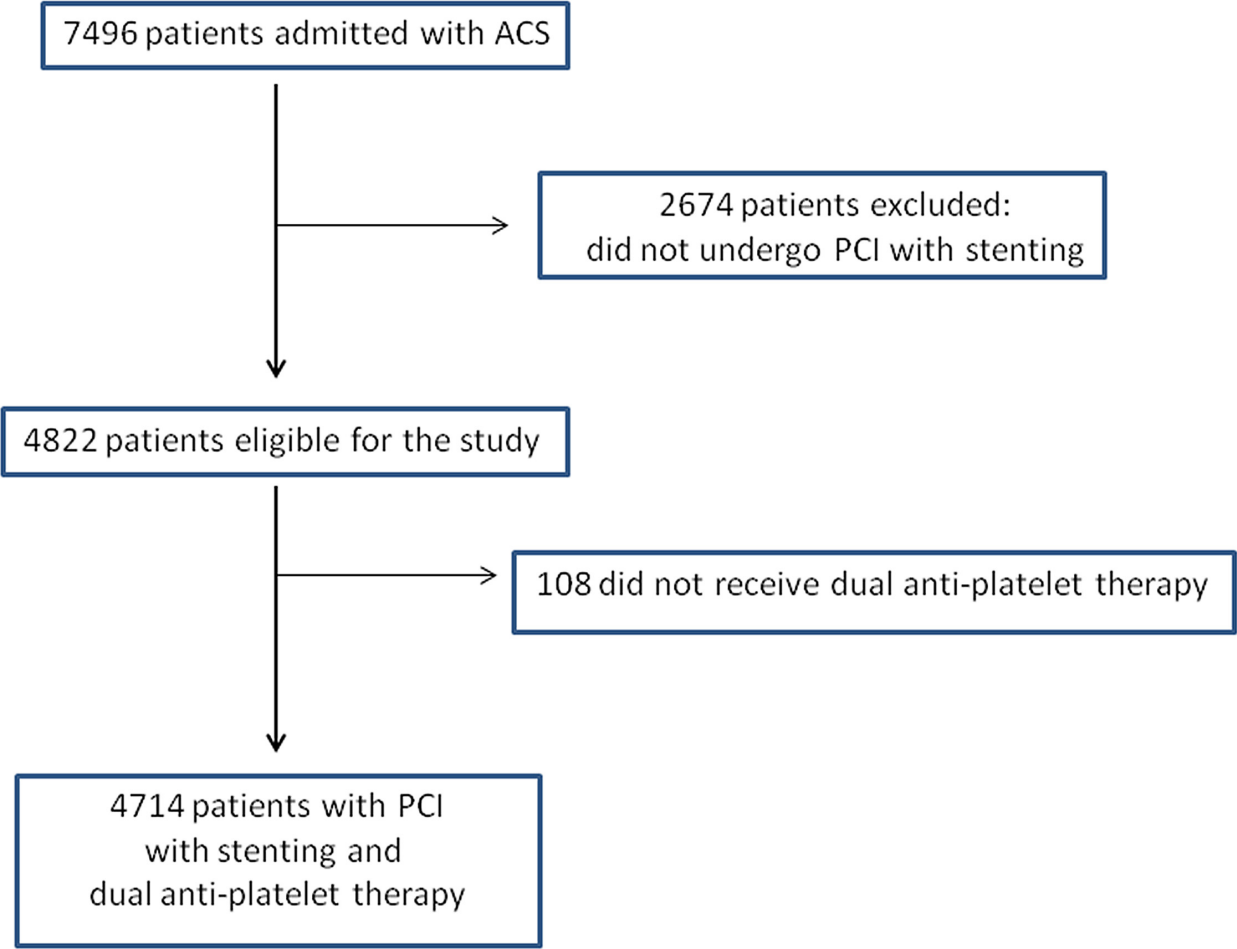
Patients' enrollment flow chart.

Patients were derived from the ACS Israeli Survey (ACSIS), a nationwide survey conducted during March and April of the years 2006, 2008, 2010 and 2013 in all 25 cardiac units and cardiology wards operating in Israel. Local ethics committee approval was received from each hospital and the study was approved by the Sheba Medical Center Institutional Review Board as well. Participants provided their written informed consent in order to participate in the study.The study population comprised all patients admitted with ACS. Patients who did not undergo PCI with stenting and who did not receive dual antiplatelet therapy were excluded from the study (Fig 1). The diagnosis of ACS was based on the presence of symptoms consistent with angina in addition to electrocardiographic changes compatible with myocardial ischemia and/or cardiac biomarker elevation. Demographic, historical, clinical and angiographic data, as well as prior medical therapy, including medications discontinued throughout the month prior to the index coronary event, were recorded on a pre-specified form for all patients. Patients were managed at the discretion of each center. All patients were either seen or contacted by telephone at 30 days post discharge. Data were collected regarding vital status, repeated procedures, including coronary angiography and/or coronary intervention, and re-hospitalization.

### Definition and endpoints

Stent thrombosis was diagnosed based on the Academic Research Consortium specifications for probable or definite stent thrombosis [8]. Stent thrombosis was defined as early (0–30 days), Late (>30 days) and very late (>12 months). Early stent thrombosis was further divided into acute (<24 hours) and sub-acute (1–30 days).

Mortality at 30 days was determined for all participants from hospital charts and by matching the identification numbers of the patients with the Israeli National Population Registry. The primary endpoint was defined as a definite early stent thrombosis, with secondary combined end points being pre-specified as all-cause mortality, recurrent ACS, stent thrombosis and/or stroke at 30 days. A new or recurrent myocardial infarction (MI) was defined as elevation of myocardial biomarkers with either repeated symptoms suggestive of ischemic and/or new ECG changes. To determine repeat MIs during the qualifying hospitalization, myocardial biomarkers were re-elevated to at least twice that of the last one measured. Recurrent ACS was defined as a recurrent MI or a recurrent ischemic event necessitating re-hospitalization or unscheduled revascularization.

### Statistical Analysis

Continuous variables are presented as mean ± SD or median and inter-quartile range, and categorical variables are expressed as percentages. Continuous variables were compared with the Student *t*-test if data followed a normal distribution and with Wilcoxon Rank sum test if data were skewed. Categorical variables were compared using chi-square test or Fisher's exact test when indicated. All tests were two-sided, and values of p<0.05 were considered statistically significant.

We used a logistic regression model in order to account for confounding baseline differences between the clopidogrel-treated patients and those receiving the newer P2Y12 inhibitors. This model included the following pre-specified variables: age (continuous), gender, hypertension, diabetes mellitus, dyslipidemia, family history of coronary artery disease (CAD), current smoking, prior stroke, chronic congestive heart failure, prior MI, prior PCI or coronary artery bypass grafting, chronic renal failure, diagnosis [STEMI vs. non-STEMI (NSTEMI)] on arrival, and P2Y12 inhibitor treatment. Statistical analysis was performed using SAS software (version 8.2, SAS Institute Inc., Cary, NC, USA).

## Results

### Baseline Characteristics

**Fig 2:**
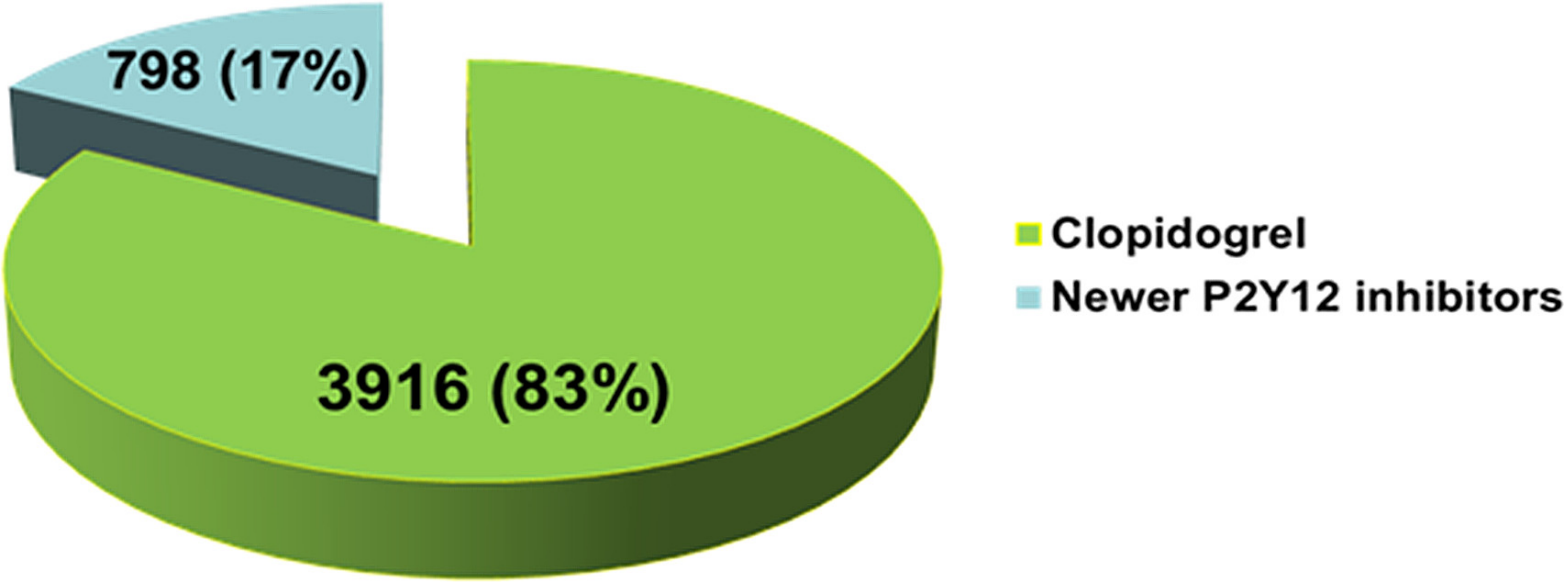
Antiplatelet drug distribution.

Of the 4714 consecutive patients with ACS who underwent PCI with stenting and received dual antiplatelet therapy in the ACSIS surveys, 3916 (83%) were treated with clopidogrel during hospitalization and 798 (17%) with the newer P2Y12 inhibitors [501 (11%) with prasugrel, and 297 (6%) with ticagrelor] (Fig 2). Baseline characteristics and prior medical therapy of the study patients are presented in Table 1. Patients who received newer P2Y12 inhibitors displayed important differences compared with those treated with clopidogrel, which included: a younger age, a higher frequency of males, STEMI at presentation, and a lower frequency of renal failure. In addition, patients receiving newer P2Y12 inhibitors tended towards a lower frequency of prior stroke and hypertension.

**Table 1:** Patients' baseline characteristics prior to medical therapy following the index ACS.

	Clopidogrel N = 3916 (83%)	Newer P2Y12 inhibitors N = 798 (17%)	P-value
Age, mean (SD), y	62 (±12)	59 (±11)	<0.01
Male, %	3144 (80%)	667 (83.5%)	0.03
STEMI	2018 (51.5%)	520 (65%)	<0.001
Dyslipidemia	2768 (71%)	558 (69.9%)	0.6
Hypertension	2288 (58.6%)	437(54.7%)	0.06
Diabetes mellitus	1273 (32%)	258 (32.3%)	0.9
Chronic renal failure	328 (8.4%)	48(6%)	<0.001
Prior MI	1006 (25.7%)	185(23.1%)	0.14
Prior PCI	1119 (28.6%)	207(25.93%)	0.14
Prior CVA/TIA	268 (6.9%)	41(5.1%)	0.08
Aspirin	1798 (46%)	310 (38.8%)	<0.003
Clopidogrel	382 (9.8%)	51(6.4%)	<0.002
Beta Blockers	1335 (34.4%)	195 (24.4%)	<0.001
ACE-I/ARBs	1424 (36.5%)	247 (31%)	<0.001
Statins	1794 (46.2%)	317 (39.7%)	<0.001

ACS. Acute coronary syndrome; SD, Standard deviation; STEMI, ST-elevation myocardial infarction; MI, Myocardial infarction, PCI, Percutaneous coronary intervention; CVA, Cerebrovascular accident; TIA, Transient ischemic attack; ACE-I, Angiotensin converting enzyme inhibitor; ARBs, Angiotensin II receptor blockers

### Early stent thrombosis

**Fig 3:**
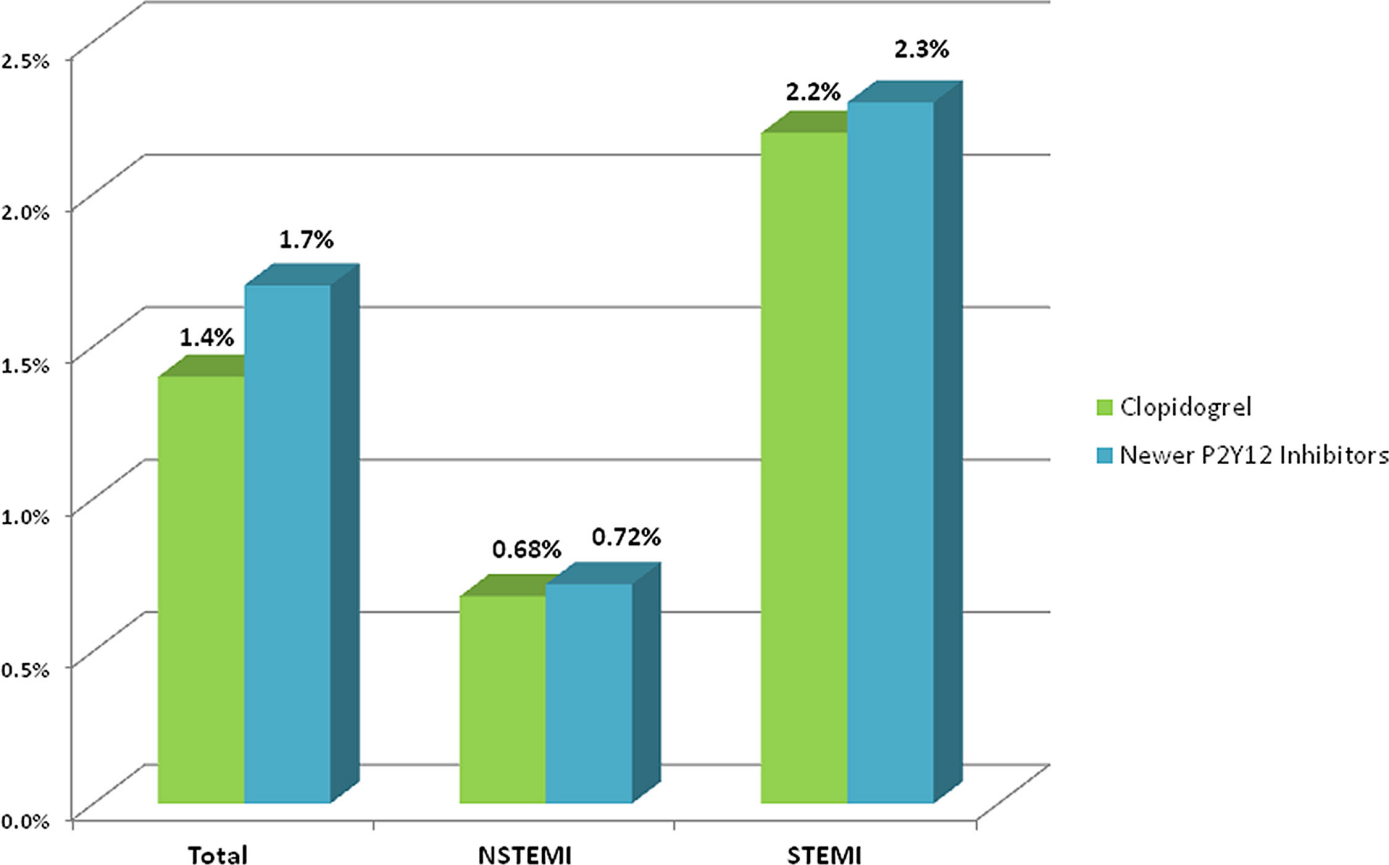
Early stent thrombosis rate.

**Fig 4:**
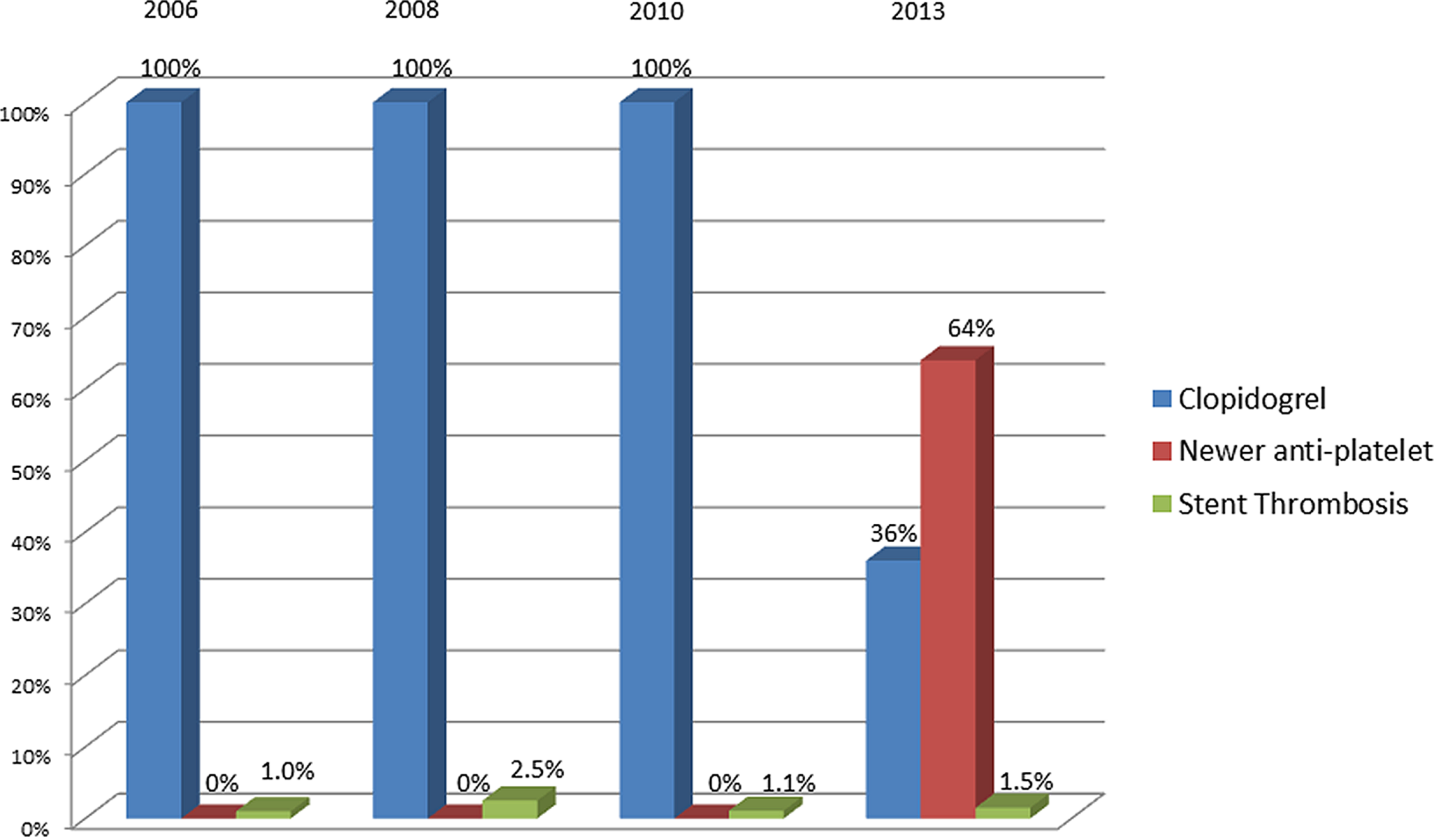
Antiplatelet distribution and stent thrombosis rate per year.

The rate of EST was similar among patients treated with newer P2Y12 inhibitors compared with clopidogrel (1.7% vs. 1.4%, respectively, p = 0.42; Fig 3). Acute and sub-acute stent thrombosis occurred in 46% vs. 56% and 54% vs. 44% with newer P2Y12 inhibitors compared with clopidogrel, respectively, p = 0.55). STEMI patients experienced a higher overall EST rate compared with other ACS patients, EST occurred at similar rates for both the newer P2Y12 inhibitors and clopidogrel (Fig 3). Moreover, the EST rate did not differ significantly throughout the years 2006, 2008, 2010 and 2013 (1%, 2.5%, 1.1%, 1.5%, respectively, P = 0.3) (Fig 4). Consistent with these univariate findings, multivariate logistic regression modeling showed that male gender and presentation with STEMI on admission were the only independent risk factors for EST, whereas treatment with newer P2Y12 inhibitors was not significantly associated with EST compared with clopidogrel therapy (adjusted HR = 1.06 [p = 0.89]) (Table 2).

**Table 2:** Multivariate logistic regression model for early stent thrombosis*.

	Odds Ratio	95% Confidence Interval	P-value
Gender (Male)	2.416	1.236–4.725	0.0099
Prior PCI	2.420	0.995–5.886	0.0514
STEMI vs. NSTEMI on presentation	5.290	2.375–11.783	<0.001

PCI, Percutaneous coronary intervention; STEMI, ST-elevation myocardial infarction; NSTEMI, Non ST-elevation myocardial infarction

* The findings were further adjusted for the following co-varieties: Age (continuous), hypertension, diabetes mellitus, dyslipidemia, family history of coronary artery disease, current smoking, prior stroke, chronic congestive heart failure, prior myocardial infarction, prior coronary artery bypass grafting, chronic renal failure, and P2Y12 inhibitors treatment.

### Major Adverse Cardiac Events

**Fig 5:**
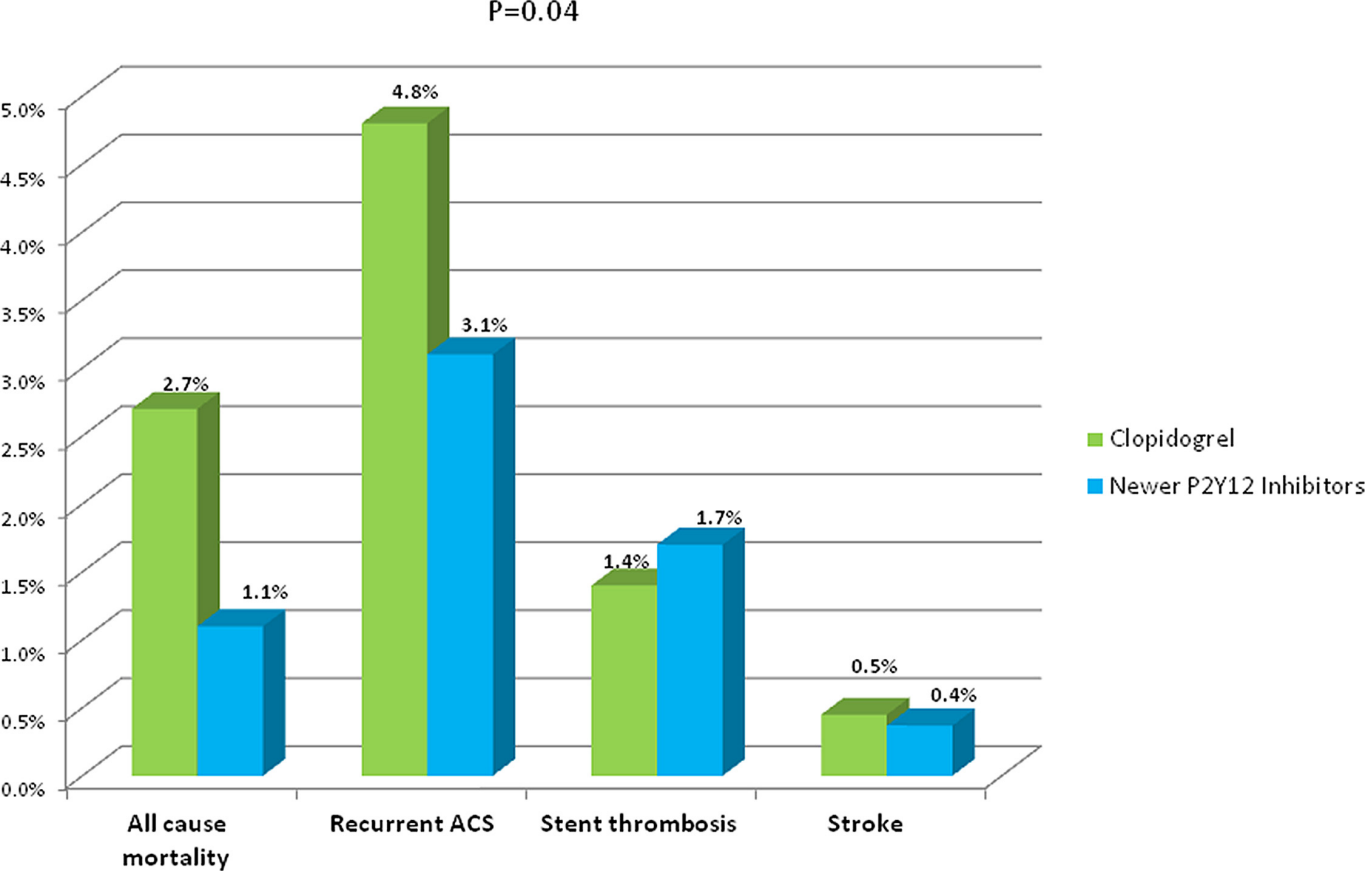
Secondary end points at 30 days.

The secondary endpoint of major adverse cardiac events (MACE) occurred in only 6.4% in the newer P2Y12 inhibitor group compared with 9.2% in the clopidogrel group (P<0.01). The difference was driven mainly by all-cause mortality (1.1% vs. 2.7%, respectively, p = 0.01) and recurrent ACS (3.1% vs. 4.8%, respectively, p = 0.04) that were both lower in the newer P2Y12 inhibitor group (Fig 5). However, multivariate logistic regression modeling showed that, after adjustment for confounders, treatment with newer P2Y12 inhibitors was not significantly associated with the secondary endpoint of MACE compared with clopidogrel therapy [OR = 1.26 95%CI (0.93–1.73), P = 0.136].

ACS=Acute coronary syndrome

Adjusted OR for overall MACE*=[1.26 95%CI(0.93-1.73),P=0.136]

* Age (continuous), gender, hypertension, diabetes mellitus, dyslipidemia, family history of coronary artery disease, current smoking, prior stroke, chronic congestive heart failure, prior myocardial infarction, prior percutaneous coronary intervention or coronary artery bypass grafting, chronic renal failure, diagnosis (ST-elevation myocardial infarction vs. non ST-elevation myocardial infarction) on arrival and P2Y12 inhibitor treatment.

## Discussion

The findings of the current study suggest that: 1) the rate of EST at 1month post-discharge in a contemporary "real life" setting is relatively low (in the range of 1.0%-1.5%); 2) over the past decade EST rates within the first month after stent implantation have remained constant despite changes in medical and interventional strategies; and 3) despite data from major randomized clinical trials favoring therapy with newer P2Y12 inhibitors over clopidogrel, our findings suggest that in a "real life" setting both 1-month EST and MACE rates might be similar in patients treated either with newer P2Y12 inhibitors or with clopidogrel. Of note, due to the relatively low rate of EST in our population (4717 patients), a larger sample may be required to show statistically significant differences between the two groups.

The only two large clinical trials to demonstrate an advantage of ticagrelor and prasugrel over clopidogrel in patients with ACS were the TRITON and PLATO trials, which enrolled more than 10,000 patients each [7, 9]. The rate of EST in our trial was in accord with the rate of EST in both these trials while in smaller clinical trials the rate of stent thrombosis was even lower [13–15]. Much like the use of newer antiplatelet therapy, others trials also sought to show a benefit of prolonged dual antiplatelet therapy in preventing stent thrombosis, but failed due to the relatively small number of patients [13–20]. The only trial that succeeded was the DAPT trial which also enrolled nearly 10,000 patients [21]. Hence, it might be that the newer antiplatelet agents have only a modest impact on the rate of stent thrombosis, and therefore a possible difference is seen only after treating thousands of patients. Even in the PLATO and TRITON trials, not only were stent thrombosis rates very low but overall event reduction rates were also low, with the number needed to treat being particularly high. Furthermore, the MACE rate was also similar in both our study groups, which again raises the question as to whether the newer antiplatelet agents provide only a modest effect on MACE. These findings are in line with the retrospective cohort analysis of the PROMETHEOUS trial [22] which examined 19,914 ACS patients, of whom 80% were treated with clopidogrel and the rest with prasugrel. The prasugrel-treated patients were younger and had less co-morbidity than those receiving clopidogrel. As in our study, after adjustment for baseline variables, MACE was similar in both groups, although in the PROMETHEOUS trial the reduction in the risk of all-cause mortality remained significant. Nevertheless, paradoxically, as in our study, bleeding rates were reduced with prasugrel in the unadjusted analysis. Another interesting point that might contribute to the modest effect of newer antiplatelet agents on stent thrombosis is their inability to change prognosis and the rate of stent thrombosis in patients non-responsive to clopidogrel. Previous data have demonstrated a strong correlation between clopidogrel non-responsiveness and adverse cardiac events which appeared to influence the incidence of stent thrombosis [10]. Moreover, an increased rate of EST was observed in non-responders compared with responders in patients scheduled for stent implantation after a 600 mg loading dose of clopidogrel [23]. Furthermore, high on-treatment platelet reactivity was found to be a strong independent predictor of stent thrombosis [24], an issue which led to the development of the newer P2Y12 inhibitors in order to overcome this limitation. However, several large clinical trials failed to show any improvement in MACE or stent thrombosis in non-responders even when the clopidogrel dose was doubled [25] or when treatment was replaced by a newer antiplatelet agent [26, 27]. Hence, changing one drug might not suffice in lowering the rate of stent thrombosis to the extent of preventing it altogether, particularly when taking into account that stent thrombosis is a multi-factorial process [28–30]. Predictors of early and late stent thrombosis following PCI with stenting have been studied in registries and post hoc analyses from clinical trials and may be categorized by: 1) the stent; 2) the patient; 3) the procedure; and 4) the type and duration of antiplatelet therapy [31–37]. It is therefore reasonable to assume that the addition of a more potent antiplatelet agent might achieve only a modest reduction in stent thrombosis rates. Moreover, a more recent study which interrogated an autopsy registry to investigate the histopathologic features of EST in patients presenting with ACS, found that the percent of necrotic core prolapse, medial tear, or incomplete apposition was significantly greater in the EST patients compared with the other patients [38]. These histopathologic features, which are prominently mechanical in nature, will probably, not be influenced by a better P2Y12 inhibitor alone.

To the best of our knowledge, this is the first study to demonstrate that the use of newer P2Y12 inhibitors is not superior to clopidogrel in terms of EST and MACE at 30 days following PCI and stent implantation in ACS patients selected from a large national registry in a “real life” setting.

### Study Limitations

Due to its observational non-randomized design, the current study is subject to limitations as described in detail previously [39]. Thus, despite efforts to control for confounding factors by applying multivariate analysis, we cannot exclude unmeasured factors which could have biased the results of the comparison between clopidogrel-treated and newer antiplatelet treated patients such as lesion characteristics and stent type and size. Another potential limitation of the study is the length of the follow-up period. Since the type of ACS (STEMI vs. unstable angina/NSTEMI) has different effects on short- and long-term prognosis, the study results regarding clinical outcomes can be applied only to short-term prognosis. Further studies are needed to examine the long-term MACE of these patients and whether newer P2Y12 inhibitors would be more beneficial in this high-risk patient subset.

## Conclusions

In summary, in this national, multicenter, contemporary “real life” setting, we did not observe any clinically meaningful differences in EST rates between clopidogrel-treated and newer antiplatelet-treated patients after PCI and stent implantation. Furthermore, it appears that throughout the past decade EST rates have been relatively low and have not changed significantly despite major changes in medical management and interventional technologies. In contrast, the rate of MACE following ACS (including mainly death and re-infarction) remains relatively high in the range of 6–9%. These findings suggest that more focus on the implementation of secondary prevention strategies is warranted in this population.

## References

[1] Mauri L, Hsieh WH, Massaro JM, Ho KK, D'Agostino R, Cutlip DE. Stent thrombosis in randomized clinical trials of drug-eluting stents. N Engl J Med. 2007; 356:1020–1029. 17296821 10.1056/NEJMoa067731

[2] Monassier JP, Hamon M, Elias J, Maillard L, Spaulding C, Raynaud P, et al Early versus late coronary stenting following acute myocardial infarction: results of the STENTIMI Study (French Registry of Stenting in Acute Myocardial Infarction). Cathet Cardiovasc Diagn. 1997; 42:243–248. 9367093 10.1002/(sici)1097-0304(199711)42:3<243::aid-ccd1>3.0.co;2-c

[3] Park DW, Park SW, Park KH, Lee BK, Kim YH, Lee CW, et al Frequency of and risk factors for stent thrombosis after drug-eluting stent implantation during long term follow-up. Am J Cardiol. 2006; 98:352–356. 16860022 10.1016/j.amjcard.2006.02.039

[4] Iakovou I, Schmidt T, Bonizzoni E, Ge L, Sangiorgi GM, Stankovic G, et al Incidence, predictors, and outcome of thrombosis after successful implantation of drug-eluting stents. JAMA 2005; 293:2126–2130. 15870416 10.1001/jama.293.17.2126

[5] Aoki J, Lansky AJ, Mehran R, Ge L, Sangiorgi GM, Stankovic G, et al Early stent thrombosis in patients with acute coronary syndromes treated with drug-eluting and bare metal stents. (The Acute Catheterization and Urgent Intervention Triage Strategy Trial). Circulation 2009; 119: 687–698. 10.1161/CIRCULATIONAHA.108.804203 19171852

[6] Beinart R, Abu Sham'a R, Segev A, Hod H, Guetta V, Shechter M, et al The incidence and clinical predictors of early stent thrombosis in patients with acute coronary syndrome. Am Heart J. 2010; 159:118–124. 10.1016/j.ahj.2009.09.020 20102877

[7] Steg PG, Harrington RA, Emanuelsson H, Katus HA, Mahaffey KW, Meier B, et al Stent thrombosis with ticagrelor versus clopidogrel in patients with acute coronary syndromes: an analysis from the prospective, randomized PLATO Trial. Circulation 2013; 128:1055–1065. 10.1161/CIRCULATIONAHA.113.002589 23900047

[8] James S, Akerblom A, Cannon CP, Emanuelsson H, Husted S, Katus H, et al Comparison of ticagrelor, the first reversible oral P2Y(12) receptor antagonist, with clopidogrel in patients with acute coronary syndromes: Rationale, design, and baseline characteristics of the PLATelet inhibition and patient Outcomes (PLATO) Trial. Am Heart J. 2009; 157:599–605. 10.1016/j.ahj.2009.01.003 19332184

[9] Wiviott SD, Braunwald E, McCabe CH, Montalescot G, Ruzyllo W, Gottlieb S, et al TRITON-TIMI 38 Investigators. Prasugrel versus clopidogrel in patients with acute coronary syndromes. N Engl J Med. 2007; 357:2001–2015. 17982182 10.1056/NEJMoa0706482

[10] Piccolo R, Di Gioia G, Niglio T, D'Anna C, De Rosa R, Strisciuglio T, et al Pharmacotherapeutic considerations for the use of prasugrel and ticagrelor to reduce stent thrombosis in patients with acute coronary syndrome. Angiology 2014; 65:130–136. 10.1177/0003319712467530 23221279

[11] Kirtane AJ, Rinaldi M, Witzenbichler B, Weisz G, Metzger DC, Henry TD, et al The increased risk of stent thrombosis in acute coronary syndromes is confined to the first 30 days after PCI. Results from the Multicenter ADAPT-DES Study. Am Coll Cardiol 2014; 64(11_S).

[12] Montalescot G, van 't Hof AW, Lapostolle F, Silvain J, Lassen JF, Bolognese L, et al ATLANTIC Investigators. Prehospital ticagrelor in ST-segment elevation myocardial infarction. N Engl J Med. 2014; 371:1016–1027. 10.1056/NEJMoa1407024 25175921

[13] Schulz-Schüpke S, Byrne RA, Ten Berg JM, Neumann FJ, Han Y, Adriaenssens T, et al Intracoronary stenting and antithrombotic regimen: Safety and efficacy of 6 months dual antiplatelet therapy after drug-eluting stenting: (ISAR-SAFE) Trial Investigators. (A randomized, double-blind, placebo-controlled trial of 6 vs. 12 months of clopidogrel therapy after drug-eluting stenting). Eur Heart J. 2015; 36:1252–1263. 10.1093/eurheartj/ehu523 25616646

[14] Park SJ, Park DW, Kim YH, Kang SJ, Lee SW, Lee CW, et al Duration of dual antiplatelet therapy after implantation of drug-eluting stents. N Engl J Med. 2010; 362:1374–1382. 10.1056/NEJMoa1001266 20231231

[15] Gilard M, Barragan P, Noryani AA, Noor HA, Majwal T, Hovasse T, et al Six-month versus 24-month dual antiplatelet therapy after implantation of drug eluting stents in patients non-resistant to aspirin. ITALIC, a randomized multicenter trial. J Am Coll Cardiol. 2015; 65:777–786. 10.1016/j.jacc.2014.11.008 25461690

[16] Costa F, Vranckx P, Leonardi S, Moscarella E, Ando G, Calabro P, et al Impact of clinical presentation on ischaemic and bleeding outcomes in patients receiving 6- or 24-month duration of dual-antiplatelet therapy after stent implantation: a pre-specified analysis from the PRODIGY—Prolonging Dual-Antiplatelet Treatment After Grading Stent-Induced Intimal Hyperplasia trial. Eur Heart J. 2015; 36:1242–1251. 10.1093/eurheartj/ehv038 25718355

[17] Gwon HC, Hahn JY, Park KW, Song YB, Chae IH, Lim DS, et al Six-month versus 12-month dual antiplatelet therapy after implantation of drug-eluting stents: the Efficacy of Xience/Promus versus Cypher to Reduce Late Loss after Stenting (EXCELLENT) randomized, multicenter study. Circulation 2012; 125:505–513. 10.1161/CIRCULATIONAHA.111.059022 22179532

[18] Kim BK, Hong MK, Shin DH, Nam CM, Kim JS, Ko YG, et al RESET Investigators. A new strategy for discontinuation of dual antiplatelet therapy: the RESET Trial (REal Safety and Efficacy of 3-month dual antiplatelet Therapy following Endeavor zotarolimus-eluting stent implantation). J Am Coll Cardiol. 2012; 60:1340–1348. 10.1016/j.jacc.2012.06.043 22999717

[19] Feres F, Costa RA, Bhatt DL, Leon MB, Botelho RV, King SB 3rd, et al Optimized duration of clopidogrel therapy following treatment with the Endeavor zotarolimus-eluting stent in real-world clinical practice (OPTIMIZE) trial: rationale and design of a large-scale, randomized, multicenter study. Am Heart J. 2012; 164: 810–816. 10.1016/j.ahj.2012.09.009 23194480

[20] Colombo A, Chieffo A, Frasheri A, Garbo R, Masotti-Centol M, Salvatella N, et al Second-generation drug-eluting stent implantation followed by 6- versus 12-month dual antiplatelet therapy. (The SECURITY randomized clinical trial). J Am Coll Cardiol. 2014; 64:2086–2097. 10.1016/j.jacc.2014.09.008 25236346

[21] Mauri L, Kereiakes DJ, Yeh RW, Driscoll-Shempp P, Cutlip DE, Steg PG, et al DAPT Study Investigators. Twelve or 30 months of dual antiplatelet therapy after drug-eluting stents. N Engl J Med. 2014; 371:2155–2166. 10.1056/NEJMoa1409312 25399658 PMC4481318

[22] Prometheous trial—Society for Cardiovascular Angiography and Interventions (SCAI) 2015 Scientific Sessions, San Diego 2015.

[23] Sibbing D, Braun S, Morath T, Mehilli J, Vogt W, Schömig A, et al Platelet reactivity after clopidogrel treatment assessed with point-of-care analysis and early drug-eluting stent thrombosis. J Am Coll Cardiol. 2009; 53: 849–856. 10.1016/j.jacc.2008.11.030 19264241

[24] Bounamici P, Marcucci R, Migliorini A, Gensini GF, Santini A, Paniccia R, et al Impact of platelet reactivity after clopidogrel administration on drug eluting stent thrombosis. J Am Coll Cardiol. 2007; 49:2312–2317. 17572245 10.1016/j.jacc.2007.01.094

[25] Price MJ, Berger PB, Teirstein PS, Tanguay JF, Angiolillo DJ, Spriggs D, et al Investigators of the GRAVITAS randomized trial. Standard- vs high-dose clopidogrel based on platelet function testing after percutaneous coronary intervention. JAMA 2011; 305:1097–1105. 10.1001/jama.2011.290 21406646

[26] Collet JP, Cuisset T, Rangé G, Cayla G, Elhadad S, Pouillot C, et al ARCTIC Investigators. Bedside monitoring to adjust antiplatelet therapy for coronary stenting. N Engl J Med. 2013; 367:2100–2109. 10.1056/NEJMoa1209979 23121439

[27] Trenk D, Stone GW, Gawaz M, Kastrati A, Angiolillo DJ, Müller U, et al A randomized trial of prasugrel versus clopidogrel in patients with high platelet reactivity on clopidogrel after elective percutaneous coronary intervention with implantation of drug-eluting stents. Results of the TRIGGER-PCI (Testing Platelet Reactivity in Patients Undergoing Elective Stent Placement on Clopidogrel to Guide Alternative Therapy with Prasugrel) study. J Am Coll Cardiol. 2012; 59:2159–2164. 10.1016/j.jacc.2012.02.026 22520250

[28] van Werkum JW, Heestermans AA, Zomer AC. Predictors of coronary stent thrombosis. The Dutch Stent Thrombosis Registry. J Am Coll Cardiol. 2009; 53:1399–1409. 10.1016/j.jacc.2008.12.055 19371823

[29] Dangas GD, Caixeta A, Mehran R, Parise H, Lansky AJ, Cristea E, et al Harmonizing Outcomes with Revascularization and Stents in Acute Myocardial Infarction (HORIZONS-AMI) Trial Investigators. Frequency and predictors of stent thrombosis after percutaneous coronary intervention in acute myocardial infarction. Circulation 2011; 123:1745–1756. 10.1161/CIRCULATIONAHA.110.981688 21482968

[30] Urban P, Gershlick AH, Guagliumi G, Guyon P, Lotan C, Schofer J, et al Safety of coronary sirolimus-eluting stents in daily clinical practice: one-year follow-up of the e-Cypher registry. Circulation 2006; 113:1434–1441. 16534015 10.1161/CIRCULATIONAHA.104.532242

[31] Spertus JA, Kettelkamp R, Vance C, Decker C, Jones PG, Rumsfeld JS, et al Prevalence, predictors, and outcomes of premature discontinuation of thienopyridine therapy after drug-eluting stent placement. Results from the PREMIER Registry. Circulation 2006; 113: 2803–2809. 16769908 10.1161/CIRCULATIONAHA.106.618066

[32] Finn AV, Joner M, Nakazawa G, Kolodgie F, Newell J, John MC, et al Pathological correlates of late drug-eluting stent thrombosis: strut coverage as a marker of endothelialization. Circulation 2007; 115: 2435–2441. 17438147 10.1161/CIRCULATIONAHA.107.693739

[33] Nakamura S, Colombo A, Gaglione A, Almagor Y, Goldberg SL, Maiello L, et al Intracoronary ultrasound observations during stent implantation. Circulation 1994; 89:2026–2034. 8181126 10.1161/01.cir.89.5.2026

[34] Goldberg SL, Colombo A, Nakamura S, Almagor Y, Maiello L, Tobis JM. Benefit of intracoronary ultrasound in the deployment of Palmaz-Schatz stents. J Am Coll Cardiol. 1994; 24: 996–1003. 7930236 10.1016/0735-1097(94)90861-3

[35] Colombo A, Hall P, Nakamura S, Almagor Y, Maiello L, Martini G, et al Intracoronary stenting without anticoagulation accomplished with intravascular ultrasound guidance. Circulation 1995; 91:1676–1688. 7882474 10.1161/01.cir.91.6.1676

[36] Grines CL, Bonow RO, Casey DE Jr, Gardner TJ, Lockhart PB, Moliterno DJ, et al Prevention of premature discontinuation of dual antiplatelet therapy in patients with coronary artery stents: a Science Advisory from the American Heart Association, American College of Cardiology, Society for Cardiovascular Angiography and Interventions, American College of Surgeons, and American Dental Association, with representation from the American College of Physicians. Circulation 2007; 115:813–818. 17224480 10.1161/CIRCULATIONAHA.106.180944

[37] Serruys PW, Morice MC, Kappetein AP, Colombo A, Holmes DR, Mack MJ, et al Percutaneous coronary intervention versus coronary-artery bypass grafting for severe coronary artery disease. N Engl J Med. 2009; 360: 961–972. 10.1056/NEJMoa0804626 19228612

[38] Nakano M, Yahagi K, Otsuka F, Sakakura K, Finn AV, Kutys R, et al Causes of early stent thrombosis in patients presenting with acute coronary syndrome: an ex vivo human autopsy study. J Am Coll Cardiol. 2014; 63:2510–2520. 10.1016/j.jacc.2014.02.607 24768883

[39] Asher E, Fefer P, Sabbag A, Herscovici R, Regev E, Mazin I, et al Prior chronic clopidogrel therapy is associated with increased adverse events and early stent thrombosis. Thromb Haemost. 2016;115(2):433–8. 10.1160/TH15-05-0384 26446379

